# *Enteromorpha prolifera* Polysaccharide Alleviates Type 2 Diabetes via the Gut Microbiota–Liver Axis to Modulate Cholesterol Metabolism

**DOI:** 10.1002/fsn3.71998

**Published:** 2026-06-09

**Authors:** Liuying Zhu, Xusheng Wen, Wenrong Zhu, Xueqian Hu, Yanqun Xu, Xin Peng

**Affiliations:** ^1^ Ningbo Municipal Hospital of Traditional Chinese Medicine (TCM) Affiliated Hospital of Zhejiang Chinese Medical University Ningbo China; ^2^ Xiangshan Xuwen Seaweed of Development Co. Ltd Ningbo China; ^3^ Department of Food Science & Technology, School of Agriculture and Biology Shanghai Jiao Tong University Shanghai China; ^4^ School of Pharmacy Zhejiang Chinese Medical University Hangzhou China

**Keywords:** cholesterol metabolism, *Enteromorpha prolifera* polysaccharide, gut microbiota, multi‐omics analyses, type 2 diabetes mellitus

## Abstract

*Enteromorpha prolifera* polysaccharide (EPP), a major bioactive sulfated polysaccharide derived from green algae, possesses potent hypoglycemic and hypolipidemic properties. This study aimed to evaluate the therapeutic efficacy of EPP in a murine model of type 2 diabetes mellitus (T2DM) and elucidate its underlying molecular mechanisms through an integrated multi‐omics approach—comprising 16S rRNA microbiomics, untargeted metabolomics, and transcriptomics. Our findings demonstrate that EPP intervention significantly suppressed fasting blood glucose (FBG) levels, attenuated dyslipidemia, and enhanced systemic insulin sensitivity. At the microbiome level, EPP restored intestinal homeostasis by enriching beneficial taxa, specifically *Lactobacillus*, *Ligilactobacillus*, and *Dubosiella*, while depleting the T2DM‐associated genus *Blautia*. These microbial shifts correlated with significantly elevated fecal concentrations of short‐chain fatty acids (SCFAs), including acetate, propionate, and branched‐chain fatty acids (isobutyrate and isovalerate). Integrated pathway analysis revealed that EPP significantly modulates steroid hormone biosynthesis; integrated pathway analysis suggested that EPP potentially modulates pathways related to steroid hormone biosynthesis, cholesterol metabolism, and primary bile acid synthesis. Quantitative RT‐PCR validation confirmed that EPP treatment was accompanied by the upregulation of critical genes involved in bile acid and steroidogenesis (*Cyp17a1*, *Cyp11a1*, *Hsd3b7*, and *Stard1*) and the downregulation of *Srebf2* and *Hnf4α*, the master transcriptional regulators of cholesterol biosynthesis and hepatic gluconeogenesis, respectively. Correlation analyses further indicated potential links between gut microbiota alterations, SCFA production, and glycemic control. Collectively, these results suggest that EPP may alleviate T2DM symptoms, which is associated with the modulation of the gut microbiota–hepatic cholesterol metabolism axis, positioning it as a promising functional food ingredient or therapeutic candidate for metabolic disorders.

## Graphical abstract

*Enteromorpha prolifera* polysaccharides may help restore lipid metabolism homeostasis by gently remodeling the gut microbiota to enrich beneficial bacteria and SCFAs, which in turn appears to modulate hepatic functions through the portal vein by downregulating cholesterol synthesis and promoting its metabolism. This coordinated process potentially leads to a reduction in liver cholesterol levels and a subtle improvement in dyslipidemia.

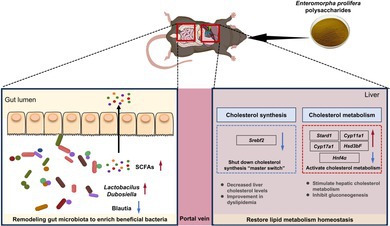

**Abbreviations**
AUC: area under curve
EPP:
*Enteromorpha prolifera* polysaccharide
ERS: endoplasmic reticulum stress
FBG: fasting blood glucose
GO: gene ontology
HbA1c: glycated hemoglobin
HDL‐C: high‐density lipoprotein‐cholesterol
H&E: hematoxylin–eosin
HOMA‐IR: insulin resistance index
KEGG: Kyoto encyclopedia of genes and genomes
LDL‐C: low‐density lipoprotein‐cholesterol
OGTT: oral glucose tolerance test
PCA: principal component analysis
PCoA: principal coordinate analysis
PLS‐DA: partial least squares discrimination analysis
QUICKI: quantitative insulin sensitivity check index
SCFAs: short‐chain fatty acids
STZ: streptozotocin
T2DM: type 2 diabetes mellitus
TC: total cholesterol
TG: total triglyceride
UPLC: ultra performance liquid chromatography

## 1 Introduction

In recent years, the accelerated pace of modernization has led to a significant increase in the global consumption of high‐fat foods. Extensive studies have demonstrated that a high‐fat diet is closely associated with the development of various metabolic disorders, including hyperlipidemia, atherosclerosis, diabetes, and dyslipidemia (Clemente‐Suarez et al. 2023). Among these conditions, type 2 diabetes mellitus (T2DM) has emerged as a critical global public health challenge. T2DM is pathologically characterized by persistent hyperglycemia, insulin resistance, and multiple complications arising from impaired insulin secretion and β‐cell dysfunction (Antar et al. 2023). Although current hypoglycemic agents exhibit certain therapeutic efficacy against diabetes, they are often accompanied by adverse effects such as hypoglycemia, edema, osteoporosis, heart failure, lactic acidosis, renal impairment, gastrointestinal disturbances, and an elevated risk of bladder cancer (Jiang et al. 2022). Consequently, the development of novel hypoglycemic drugs or adjuvant therapies derived from natural products holds significant clinical importance.

Polysaccharides have demonstrated remarkable bioactivities in regulating lipid metabolism, controlling blood glucose levels, and ameliorating lipid metabolism disorders (Xinmei Xu, Wang, et al. 2023). Currently, various polysaccharide‐based preparations have been developed and applied in the clinical treatment of T2DM (Bo et al. 2024). *Enteromorpha prolifera* , a macroalga belonging to the phylum Chlorophyta and class Ulvophyceae, is widely distributed in coastal regions worldwide (Huang et al. 2019). This algal species is rich in carbohydrates and high‐quality proteins, characterized by its high‐protein, low‐fat, and unsaturated fatty acid‐rich nutritional profile, making it a valuable marine functional food resource (J. Xu, Liao, et al. 2023). Research has revealed that EPP, as the primary active components in the alga, possess multiple pharmacological activities including immunomodulatory, antibacterial, antiviral, antioxidant, antitumor, anti‐inflammatory, and moisturizing effects (Tang et al. 2013; Wassie et al. 2021). These findings collectively indicate that EPP exhibits multiple bioactivities such as antioxidant, antitumor, and lipid metabolism regulation, suggesting its potential as a promising resource for developing hypoglycemic and hypolipidemic agents. However, current research on the hypoglycemic effects of EPP remains relatively limited, and its precise mechanisms of action require further elucidation.

Recent advances in high‐throughput sequencing and mass spectrometry technologies have revolutionized systems biology, enabling comprehensive integration of diverse biological components for investigating complex physiological processes (Dai and Shen 2022). The emerging multi‐omics integration approach has proven particularly powerful in deciphering intricate biomolecular networks (Vitorino 2024). In this study, we employed an integrated multi‐omics strategy combining gut microbiomics, transcriptomics, and metabolomics to systematically investigate the potential mechanisms underlying the protective effects of EPP against high‐fat diet‐induced T2DM in mice. Our findings not only elucidate the hypoglycemic mechanisms of EPP but also provide a scientific foundation for its potential application in functional food development.

## 2 Materials and Methods

### 2.1 Extraction and Purification of EPP

*Enteromorpha prolifera* collected from Ningbo, Zhejiang Province, China, was used in this study. Crude polysaccharides were extracted from 150 g of algal powder (solid‐to‐liquid ratio 1:10) by hot water extraction (100°C, 2 h), repeated twice. The combined extracts were centrifuged at 4000 rpm for 10 min, and the supernatant was concentrated to 1/5 of its original volume. Ethanol was added to the concentrated solution to a final concentration of 80% (v/v) for polysaccharide precipitation (4°C, 12 h). The precipitate was collected by centrifugation (4000 rpm, 10 min) to obtain crude polysaccharides. For protein removal, 10 g of crude polysaccharides were dissolved in ultrapure water (1:100, g/mL), and 1 g of neutral protease (200,000 U/g) was added. The solution was incubated at 55°C for 2 h, followed by protease inactivation in a 100°C water bath for 10 min. After centrifugation (4000 rpm, 10 min), the supernatant was dialyzed using a 3.5 kDa molecular weight cutoff membrane for 48 h. The dialyzed solution was concentrated and lyophilized to obtain purified EPP.

### 2.2 Composition Analysis of EPP

A comprehensive analysis of the chemical composition of EPP was conducted in this study. The total sugar content was determined by the phenol–sulfuric acid method, while the protein content was measured using a Solarbio Bradford protein assay kit (Beijing, China). Monosaccharide composition analysis was performed on a Thermo ICS 5000+ ion chromatography system (Thermo Fisher Scientific, USA) equipped with an electrochemical detector for accurate identification of individual monosaccharides. To characterize the structural features of EPP, the dried sample was mixed with potassium bromide at a 1:200 (w/w) ratio, ground thoroughly, and pressed into a 1 mm‐thick pellet for Fourier‐transform infrared spectroscopy (Frontier, PerkinElmer) scanning within the 4000–400 cm^−1^ wavelength range to obtain the infrared absorption spectrum. The homogeneity and molecular weight of various fractions were assessed using Size Exclusion Chromatography coupled with Multi‐Angle Light Scattering and Refractive Index detection (SEC‐MALLS‐RI). The weight‐average molecular weight (Mw), number‐average molecular weight (Mn), and polydispersity index (Mw/Mn) of the different fractions in a 0.1 M NaNO_3_ aqueous solution containing 0.02% sodium azide (NaN_3_) were measured utilizing a DAWN HELEOS‐II laser photometer (Wyatt Technology Co., USA). The molecular weight (Mw, Mn) and polydispersity (Mw/Mn) of polysaccharide fractions were determined using a SEC‐MALS‐RI system. The analysis was performed on a DAWN HELEOS‐II laser photometer coupled with an Optilab T‐rEX refractive index detector (Wyatt Technology, USA). Separation was achieved using two tandem columns (Shodex OH‐pak SB‐805 and SB‐803) at 45°C. The mobile phase consisted of 0.1 M NaNO_3_ (containing 0.02% NaN_3_) at a flow rate of 0.6 mL/min. A dn/dc value of 0.141 mL/g was utilized for calculations. Data were acquired and processed using ASTRA 6.1 software (Wyatt Technology).

### 2.3 Animals and Treatments

50 male C57BL/6 mice (4‐week‐old, 18–22 g body weight) purchased from Vital River Laboratory Animal Technology Co. Ltd. (Beijing, China) were used in this study. As illustrated in Figure 2A, after 1 week of acclimatization, the mice were randomly divided into two parts: normal control group (NC, *n* = 8, fed with standard diet D12450) and high‐fat/high‐sucrose diet group (*n* = 40, fed with 60% fat diet D12492). After 4 weeks of dietary intervention, following a 12‐h fasting period, the mice received intraperitoneal injection of freshly prepared streptozotocin (STZ) solution (100 mg/kg in 0.1 mol/L citrate buffer, pH 4.5), while the control group received equivalent volume of citrate buffer. After 72 h, fasting blood glucose (FBG) was measured using a glucometer, with FBG ≥ 11.1 mmol/L indicating successful diabetes induction. Of the 42 mice in the high‐fat/high‐sucrose diet group, 22 mice failed to meet this criterion after the first STZ injection and received an additional dose of STZ (50 mg/kg). After the second injection, 20 mice achieved FBG ≥ 11.1 mmol/L, with 2 mice requiring exclusion. No mice died during the modeling process. Consequently, a total of 40 mice were successfully modeled. The successfully modeled mice were then randomly allocated into five groups: model control group (MC, *n* = 8), Met group (*n* = 8, metformin 250 mg/kg), EPP‐L group (*n* = 8, EPP 50 mg/kg), EPP‐M group (*n* = 8, EPP 100 mg/kg), and EPP‐H group (*n* = 8, EPP 200 mg/kg). No mice died during the administration period, resulting in final sample sizes of *n* = 8 for all groups. Daily oral gavage administration was performed for 5 consecutive weeks, with weekly body weight monitoring. The experimental protocol was approved by the Animal Ethics Committee of Guoke Ningbo Life Science and Health Industry Research Institute (Approval No. GK‐2025‐XM‐0003), and all procedures strictly adhered to ethical guidelines for laboratory animals. The dose of EPP‐H (200 mg/kg) was selected for mechanistic studies based on our preliminary dose–response studies, which demonstrated its optimal efficacy in improving glucose tolerance and lipid profiles compared with lower doses. This dosage also aligned with the human equivalent dose calculated by body surface area normalization and was consistent with previous pharmacological studies on EPP (Zhu et al. 2023).

### 2.4 Fasting Blood Glucose and Insulin Assays

This study employed comprehensive methods for metabolic parameter detection. Fasting blood glucose levels were measured via tail vein sampling using a Roche glucometer and compatible test strips, with plasma glucose concentrations further quantified by glucose oxidase assay (glucose diagnostic kit, Jiancheng, Nanjing, China) after 5 weeks of gavage treatment. Fasting insulin was detected by two‐antibody sandwich ELISA: serum samples and standards were added to insulin antibody‐coated microplates followed by HRP‐conjugated secondary antibody incubation at 37°C; after thorough washing, TMB substrate was added to generate a blue product catalyzed by HRP, which turned yellow after acid stop solution addition; absorbance at 450 nm was finally measured using a microplate reader for insulin concentration calculation based on the standard curve.

### 2.5 Assessment of Insulin Sensitivity

To evaluate insulin sensitivity in experimental diet‐fed mice, we calculated two well‐established indices: homeostasis model assessment of insulin resistance (HOMA‐IR) and Quantitative insulin sensitivity check index (QUICKI); the indices were calculated as follows:

$$\text{HOMA}-\mathrm{IR}=\left[\text{fasting insulin}\;\left(\mathrm{\mu IU}/\mathrm{mL}\right)\times \text{fasting glucose}\;\left(\text{mmol}/L\right)\right]/22.5$$

$$\text{QUICKI}=1/\left[\log\;\left(\text{fasting insulin}\;\left(\mathrm{\mu IU}/\mathrm{mL}\right)\right)+\log\;\left(\text{fasting glucose}\;\left(\mathrm{mg}/\mathrm{dL}\right)\right)\right]$$

### 2.6 Oral Glucose Tolerance Test

An oral glucose tolerance test (OGTT) was performed during the final experimental week. After 12 h of fasting, mice were administered glucose solution (1.5 g/kg body weight) by gavage. Blood glucose levels were measured via tail vein sampling at baseline (0 min) and at 15, 30, 60, 90, and 120 min post‐administration. Glucose tolerance was evaluated by calculating the area under the curve (AUC) to comprehensively assess glucose metabolism function.

### 2.7 Serum Biochemical Analysis

Comprehensive serum biochemical analysis was conducted in this study. Whole blood samples were first kept at 4°C for 2 h, followed by centrifugation at 4000 rpm for 20 min to obtain serum. The levels of glycosylated hemoglobin A1C (HbA1c), triglyceride (TG), total cholesterol (TC), high‐density lipoprotein cholesterol (HDL‐C), and low‐density lipoprotein cholesterol (LDL‐C) were determined using specific commercial assay kits (Jiancheng, Nanjing, China), with all measurements performed and analyzed on a Multiskan MK3 microplate reader (Thermo Fisher Scientific, USA).

### 2.8 Histopathological Analysis

A systematic histopathological examination was performed on liver tissues. Immediately after collection, the hepatic tissues were rinsed with ice‐cold physiological saline and fixed in 4% paraformaldehyde at 25°C for 48 h. Following dehydration through a graded ethanol series, the samples were embedded in paraffin and sectioned at 4 μm thickness. The sections were baked at 65°C for 6 h to enhance tissue adhesion and subsequently stained with hematoxylin and eosin (H&E). All tissue sections were examined and imaged using a Nikon inverted microscope (Nikon TS2, Japan), and the acquired digital images were analyzed and processed with Case Viewer software (3DHISTECH Ltd., Hungary).

### 2.9 Quantitative Real‐Time PCR Analysis

Gene expression levels in liver tissues were determined by quantitative real‐time PCR (qRT‐PCR). Total RNA was extracted from liver tissues using Trizol reagent (Invitrogen, Carlsbad, CA), followed by reverse transcription of 1 μg RNA into cDNA using a Takara reverse transcription kit (Takara, Japan). The PCR reaction mixture (10 μL total volume) consisted of 5 μL SYBR Green master mix, 0.5 μL each of forward and reverse primers (sequences listed in Table S1), 2 μL cDNA template, and 2 μL nuclease‐free water (ddH2O). The thermal cycling conditions were: initial denaturation at 95°C for 30 s, followed by 35 cycles of 95°C for 5 s and 60°C for 30 s. All reactions were performed on a Bio‐Rad real‐time PCR system (Bio‐Rad, Hercules, CA, USA), with β‐actin as the internal reference gene. Relative mRNA expression levels were calculated using the 2^−ΔΔCt^ method. Primer sequences were shown in Table S1.

### 2.10 Short‐Chain Fat Acids Analysis

For the analysis of short‐chain fat acids (SCFAs) in fecal samples, sample preparation involved adding 50 μL of 30% phosphoric acid solution and 300 μL of acetone to the fecal sample, followed by homogenization at 12,000 rpm for 3 min and centrifugation for 10 min. The resulting supernatant was collected and diluted as required prior to instrumental analysis. Separation and quantification were performed using an Agilent 7820 gas chromatography system (Agilent Technologies, USA) equipped with a DB‐FFAP capillary column (30 m × 0.25 mm × 0.25 μm). Chromatographic conditions included a 1 μL injection volume with a 10:1 split ratio, high‐purity helium carrier gas at a constant flow rate of 1.0 mL/min, and a temperature program initiating at 70°C (held for 5.0 min) followed by a ramp to 100°C at 6°C/min.

### 2.11 Liver Transcriptome Analysis

Liver transcriptome sequencing was performed by Biomarker Technologies (Beijing, China) with five biological replicates per group. Total RNA was extracted using TRIzol reagent (Life Technologies, USA), followed by quality assessment with NanoDrop 2000 (A260/A280 ratio 1.8–2.1) and Agilent 2100 Bioanalyzer/LabChip GX (RIN ≥ 7.0). After polyA selection, strand‐specific cDNA libraries were prepared using NEBNext Ultra II RNA Library Prep Kit. Library quality control included Qubit 3.0 fluorometric quantification (≥ 1 ng/μL), Qsep400 analysis (insert size 200–500 bp), and qPCR quantification (effective concentration > 2 nM). Sequencing was performed on Illumina NovaSeq 6000 platform (PE150 mode, 20 million clean reads/sample). Raw data were processed through BMKCloud (www.biocloud.net) for quality control, alignment (STAR v2.7.9a), and quantification (RSEM v1.3.3). Differential expression analysis using DESeq2 (v1.39.0) identified differential expressed genes with |log2FoldChange| ≥ 0.585 (fold change ≥ 1.5) and FDR‐adjusted *p*‐value < 0.05. Functional enrichment analysis was conducted using cluster Profiler (v4.4.4) for Gene Ontology (GO) and Kyoto Encyclopedia of Genes and Genomes (KEGG) analyses (significance threshold *p* adjust < 0.05).

### 2.12 Liver Metabolomics Analysis

The untargeted metabolomics analysis of liver tissues was conducted by Biomarker Technologies (Beijing, China) with six biological replicates per group. Metabolite extraction was performed using a mechanical grinding‐assisted organic solvent method, where precisely weighed liver tissues were homogenized with optimized extraction solvent and zirconia beads, followed by ultrasonic‐assisted extraction, low‐temperature incubation, centrifugation, and vacuum concentration of the supernatant prior to reconstitution for analysis. The instrumental analysis was carried out on a Waters Acquity I‐Class PLUS UHPLC system coupled with a Waters Xevo G2‐XS QTOF high‐resolution mass spectrometer, using an ACQUITY UPLC HSS T3 column (2.1 × 100 mm, 1.8 μm) maintained at 40°C, with electrospray ionization (ESI) in both positive and negative modes scanning from 50 to 1200 m/z. Raw data acquired by MassLynx V4.2 were processed using Progenesis QI software for peak picking and alignment, with metabolite identification against both the METLIN database and Biomark in‐house library (mass error threshold < 100 ppm). Multivariate statistical analyses including principal component analysis (PCA) and principal coordinate analysis (PCoA) were employed to identify significantly altered metabolites (VIP > 1 and *p* < 0.05), followed by pathway enrichment and topological analysis using the MetaboAnalyst platform.

### 2.13 16S rRNA Sequencing of Gut Microbiota

Microbial genomic DNA was extracted from colon contents using TGuide S96 Magnetic Stool DNA Kit (Tiangen Biotech, China). The V3‐V4 hypervariable regions of bacterial 16S rRNA genes were amplified with primers 338F (5′‐ACTCCTACGGGAGGCAGCA‐3′) and 806R (5′‐GGACTACHVGGGTWTCTAAT‐3′). PCR products were visualized by agarose gel electrophoresis and purified with Omega DNA Purification Kit (Omega Inc., USA). Purified amplicons were sequenced on Illumina Novaseq 6000 platform (2 × 250 bp paired‐end). After quality control, sequences were clustered into OTUs at 97% similarity threshold using USEARCH (v10.0) and taxonomically annotated against SILVA database (v138.1) with Naive Bayes classifier in QIIME2 (confidence threshold 70%). Alpha diversity (Chao1, Shannon, Simpson indices) was calculated to assess within‐sample diversity, while beta diversity was evaluated by PCoA and partial least squares discrimination analysis (PLS‐DA). Line discriminant analysis effect size analysis (LEfSe, LDA score > 2.0, *p* < 0.05) identified differentially abundant taxa, with all analyses performed on BMKCloud platform (https://www.biocloud.net).

### 2.14 Integration of Metabolome and Gut Microbiota Data Using WGCNA

To explore associations between liver metabolites and gut bacterial genera, weighted gene coexpression network analysis (WGCNA) was conducted with default parameters via the BMKCloud Platform (https://international.biocloud.net). Metabolic modules and trait‐related networks were constructed, and a network heatmap along with module visualizations were generated. Low‐abundance metabolites (SD < 0.5) were excluded to focus on meaningful biological variation. Module–trait relationship analysis based on Pearson correlation was used to identify bacterial genera significantly linked to metabolic profiles. Correlations with *p*‐values below 0.05 were considered statistically significant and selected for further analysis.

### 2.15 Correlation Analysis Between Gut Microbiota and Biochemical Parameters

Spearman correlation analyses were conducted using OriginPro to analyze the correlations among gut microbiota, SCFAs, and serum parameters, including FBG, AUC, HOMA‐IR, IFCC‐HbA1c, TG, TC, LDL‐C, HDL‐C, and QUICKI in each mice group.

### 2.16 Statistical Analysis

All data are presented as the mean ± standard error of the mean (SEM). Statistical analyses were performed using GraphPad Prism software (version 9.0) or SPSS (version 26.0). For static parameters measured at a single time point (e.g., terminal serum biochemical markers, organ weights, and SCFA levels), a one‐way analysis of variance (ANOVA) followed by Tukey's post hoc test or Duncan's multiple range test was employed to determine significance between multiple groups.

For longitudinal data involving multiple time points, including weekly body weight, FBG monitoring, and glucose concentrations during the OGTT, a two‐way repeated measures ANOVA was conducted, with ‘treatment’ as the between‐subject factor and ‘time’ as the within‐subject factor. Post hoc comparisons at specific time points were performed using Sidak's or Bonferroni's multiple comparisons test. A *p*‐value of < 0.05 was considered statistically significant.

For microbiome and metabolomics datasets, the Benjamini‐Hochberg procedure was applied to control the false discovery rate (FDR) for multiple‐testing comparisons. Unless otherwise specified, a *p*‐value of < 0.05 was considered statistically significant.

## 3 Results

### 3.1 Chemical Composition and Structural Characterization of EPP

**FIGURE 1:**
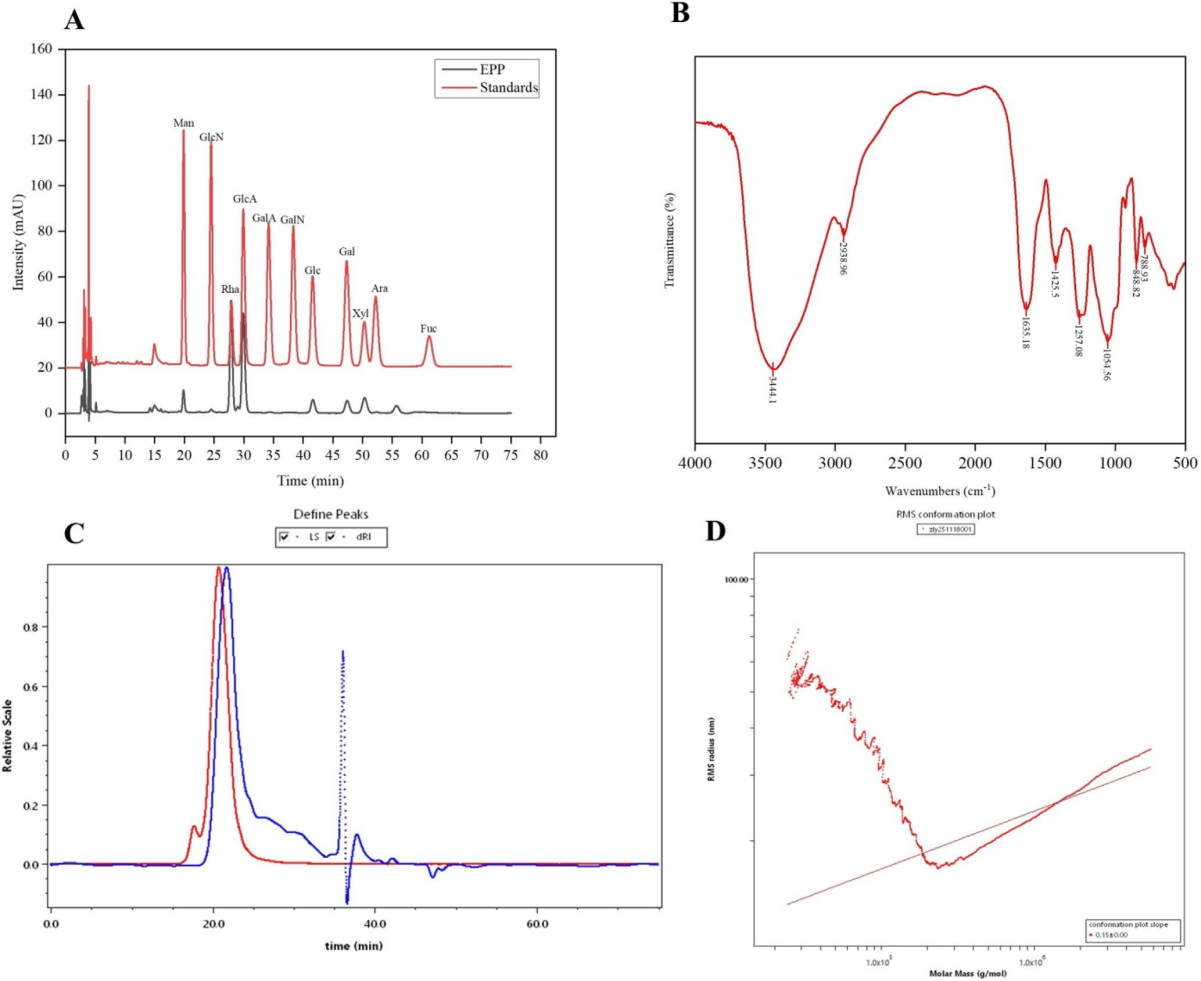
Chemical characterization of EPP. (A) HPLC profiles of the monosaccharide composition of the standards and EPP; (B) FTIR spectrum of EPP; (C) molecular weight analysis plot; (D) molecular configuration diagram. Ara, Arabinose; Fru, Fructose; Gal, Galactose; GalA, Galacturonic acid; GalN, Galactosamine; Glc, Glucose; GlcA, Glucuronic acid; GlcN, Glucosamine; Man, Mannose; Rha, Rhamnose; Xyl, Xylose.

The total carbohydrate content of EPP was determined to be 69.07% ± 0.62%, while its protein content was measured at 1.95% ± 0.58%. Monosaccharide composition analysis revealed that EPP contained rhamnose (Rha), glucuronic acid (GlcA), xylose (Xyl), mannose (Man), glucosamine (GlcN), glucose (Glc), galactose (Gal), and arabinose (Ara) at a molar ratio of 57.84:18.97:12.29:2.49:0.71:4.05:3.42:0.22 (Figure 1A).

The molecular weight and chain conformation of EPP were investigated using SEC‐MALS‐RI (Figure 1C). EPP exhibited a single, relatively symmetrical peak in both RI and LS signals, indicating its homogeneity. The Mw was determined to be 415.97 kDa with a polydispersity index (Mw/Mn) of 4.658. Notably, the conformation plot (Figure 1D), which depicts the relationship between the root‐mean‐square (RMS) radius and molar mass, yielded a slope of 0.15 ± 0.00. This low slope value (lower than 0.33) suggests that EPP likely adopts a highly compact, hyperbranched, or spherical‐like conformation in 0.1 M NaNO_3_ solution.

The functional groups of EPP were identified by FTIR spectroscopy (Figure 1B). A strong, broad peak at 3444.10 cm^−1^ was attributed to O—H stretching vibrations from intermolecular or intramolecular hydrogen bonding. A medium‐intensity, sharp peak near 2938.96 cm^−1^ originated from C—H stretching vibrations of methyl (—CH_3_) or methylene (—CH_2_) groups. The peak at 1635.18 cm^−1^ was assigned to the H—O—H bending vibration of water molecules. Peaks at 1425.50 and 1257.08 cm^−1^ corresponded to C—H in‐plane bending vibrations characteristic of sugar rings. The strong peak at 1054.56 cm^−1^, resulting from C—O stretching vibrations of C—O—C or C—O—H bonds, is a key indicator of the pyranose ring structure. Significantly, the peak at 848.82 cm^−1^ demonstrated the C—H out‐of‐plane bending vibration specific to α‐anomeric pyranose sugars, providing clear evidence for an α‐glycosidic linkage configuration, while the peak at 788.93 cm^−1^ corresponded to the out‐of‐plane deformation vibration of the pyranose ring skeleton, further confirming the presence of six‐membered sugar rings.

### 3.2 EPP Improves Glycemic Control and Insulin Sensitivity in Type 2 Diabetic Mice

**FIGURE 2:**
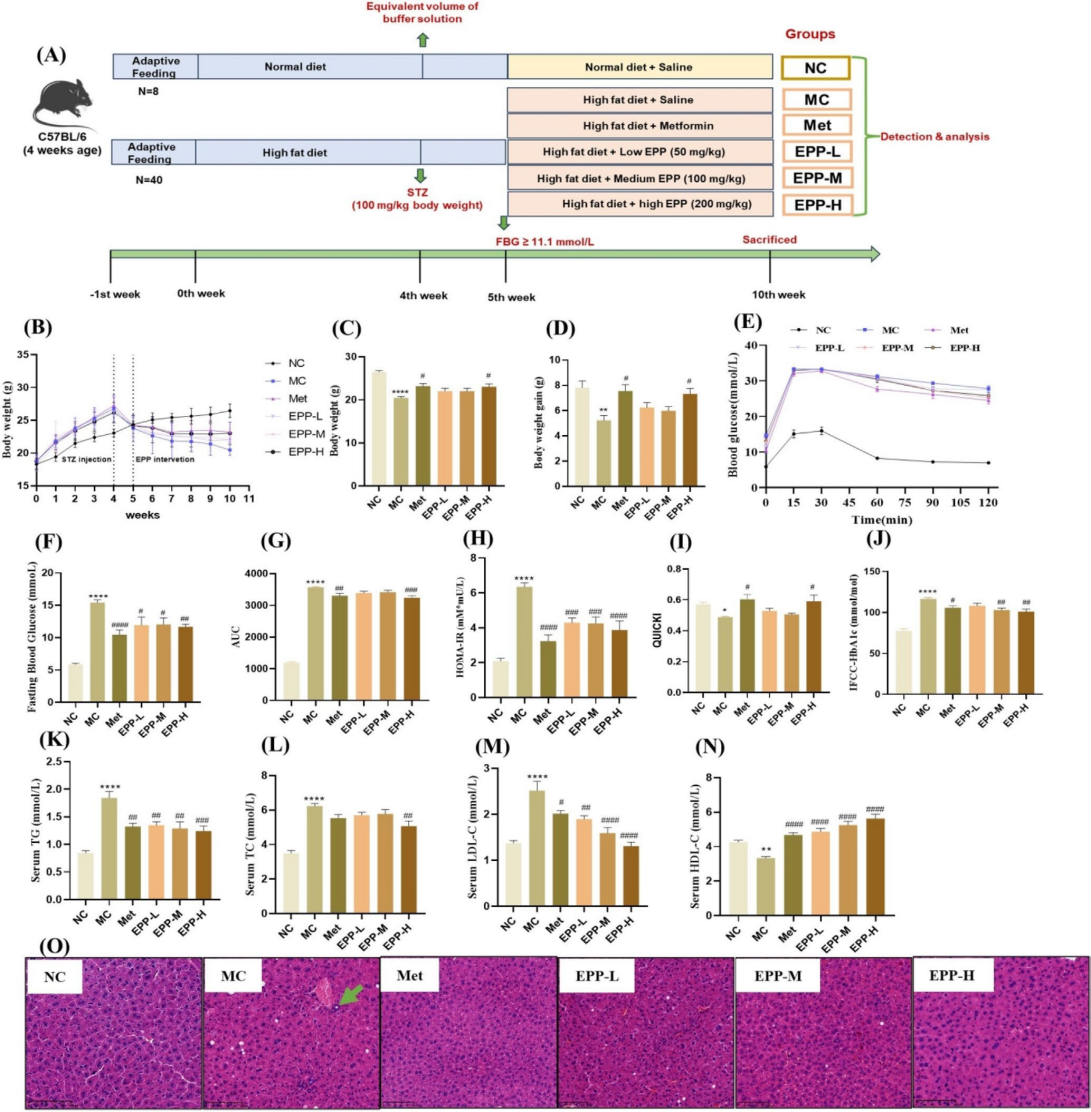
Beneficial effects of EPP on glycemic regulation. (A) Workflow of the T2DM mouse model establishment and EPP intervention. (B) Body weight changes during the trial. (C) Body weight at the end of the experiment. (D) Body weight gain. (E) Oral glucose tolerance test (OGTT). (F) Fasting blood glucose (FBG). (G) Area under the curve (AUC) of OGTT. (H) Homeostasis model assessment of insulin resistance (HOMA‐IR). (I) Quantitative insulin sensitivity check index (QUICKI). (J) Glycosylated hemoglobin (HbA1c). (K) Serum TG. (L) Serum TC. (M) Serum LDL‐C. (N) Serum HDL‐C. (O) Liver histopathology (H&E staining, scale bar = 100 μm). HDL‐C, high‐density lipoprotein cholesterol; LDL‐C, low‐density lipoprotein cholesterol; MC, model control; NC, normal control; TC, total cholesterol; TG, triglycerides. *Statistical analysis:* Data are expressed as mean ± SEM (*n* = 8). Two‐way repeated measures ANOVA with Sidak's *post hoc* test was used for longitudinal data (B and E), while one‐way ANOVA with Tukey's test was used for other parameters. Compared with the NC group, *p < 0.05, **p < 0.01, ****p < 0.0001; compared with the MC group, ^#^ *p* < 0.05, ^##^ *p* < 0.01, ^###^ *p* < 0.001, ^####^ *p* < 0.0001.

This study systematically evaluated the therapeutic effects of EPP on type 2 diabetic mice. Compared with NC group, MC group showed significant body weight loss (Figure 2B,C, *p* < 0.01), which was effectively reversed by metformin (Met) and EPP treatments (Figure 2C). For glycemic parameters, FBG in MC group was markedly elevated (Figure 2F, *p* < 0.0001). Both Met and EPP interventions significantly reduced FBG levels, with EPP‐H group achieving a 31.8% reduction (*p* < 0.01). OGTT revealed severe glucose intolerance in MC group (*p* < 0.0001), which was significantly ameliorated by Met and EPP treatments, particularly in the EPP‐H group (Figure 2G, *p* < 0.001). These results clearly demonstrate that EPP significantly improved glucose intolerance in diabetic mice, with EPP‐H showing efficacy similar to Met.

Further evaluation of insulin sensitivity revealed that the HOMA‐IR value in the MC group increased approximately 3.1‐fold compared to the NC group (Figure 2H, *p* < 0.0001). Both Met and EPP administration significantly reduced this value, with the EPP‐H group showing a 39.1% reduction (*p* < 0.0001), comparable to the Met‐positive control. Additionally, the QUICKI level in the MC group was markedly lower than that in the NC, Met, and EPP‐H groups (Figure 2I, *p* < 0.05). Furthermore, EPP treatment significantly attenuated the elevated glycated hemoglobin levels in diabetic mice, with EPP‐M and EPP‐H demonstrating the most pronounced effect (Figure 2J, *p* < 0.01). Given the superior efficacy of EPP‐H, which was comparable to or even surpassed Met in some parameters, subsequent mechanistic studies will focus on the high‐dose EPP group.

### 3.3 EPP Ameliorates Dyslipidemia and Hepatic Steatosis in T2DM Mice

To assess the impact of EPP on T2DM, we measured serum TG, TC, LDL‐C, and HDL‐C levels, along with histological examination via H&E staining. Compared to the control group, the MC group exhibited significantly elevated serum TG, TC, and LDL‐C levels (Figure 2K–M, *p* < 0.0001), accompanied by reduced HDL‐C levels (Figure 2N, *p* < 0.01). EPP treatments markedly decreased TG levels (Figure 2K, *p* < 0.01), with EPP‐H showing the most substantial reduction and demonstrating clear dose‐dependent effects on both TC and LDL‐C (Figure 2L,M). Moreover, HDL‐C levels (*p* < 0.0001) were significantly upregulated following Met and EPP administration compared to the MC group, with EPP‐H exhibiting the most prominent improvement.

Given the close association between hepatic injury and T2DM, we further evaluated the hepatoprotective effects of EPP. Histopathological analysis of H&E‐stained liver sections revealed pronounced central venous dilation, hepatocellular hypertrophy, and disorganized hepatic plate architecture in the MC group. Additionally, hepatocyte degeneration was evident, characterized by extensive lipid vacuolation and disruption of normal hepatic structure, indicating significant triglyceride accumulation and pathological alterations in T2DM mice (Figure 2O). Strikingly, both Met and EPP interventions substantially ameliorated these hepatic abnormalities, with EPP‐H showing the most remarkable reduction in lipid deposition and restoration of tissue morphology. Collectively, these findings demonstrate that EPP effectively mitigates hepatic injury and ameliorates dyslipidemia in T2DM mice in a dose‐dependent manner, with EPP‐H exhibiting the strongest therapeutic potential comparable to Met, warranting its selection for subsequent mechanistic investigations.

### 3.4 EPP Ameliorates Diabetes‐Induced Gut Dysbiosis by Modulating Microbial Composition and Promoting Probiotic Growth

**FIGURE 3:**
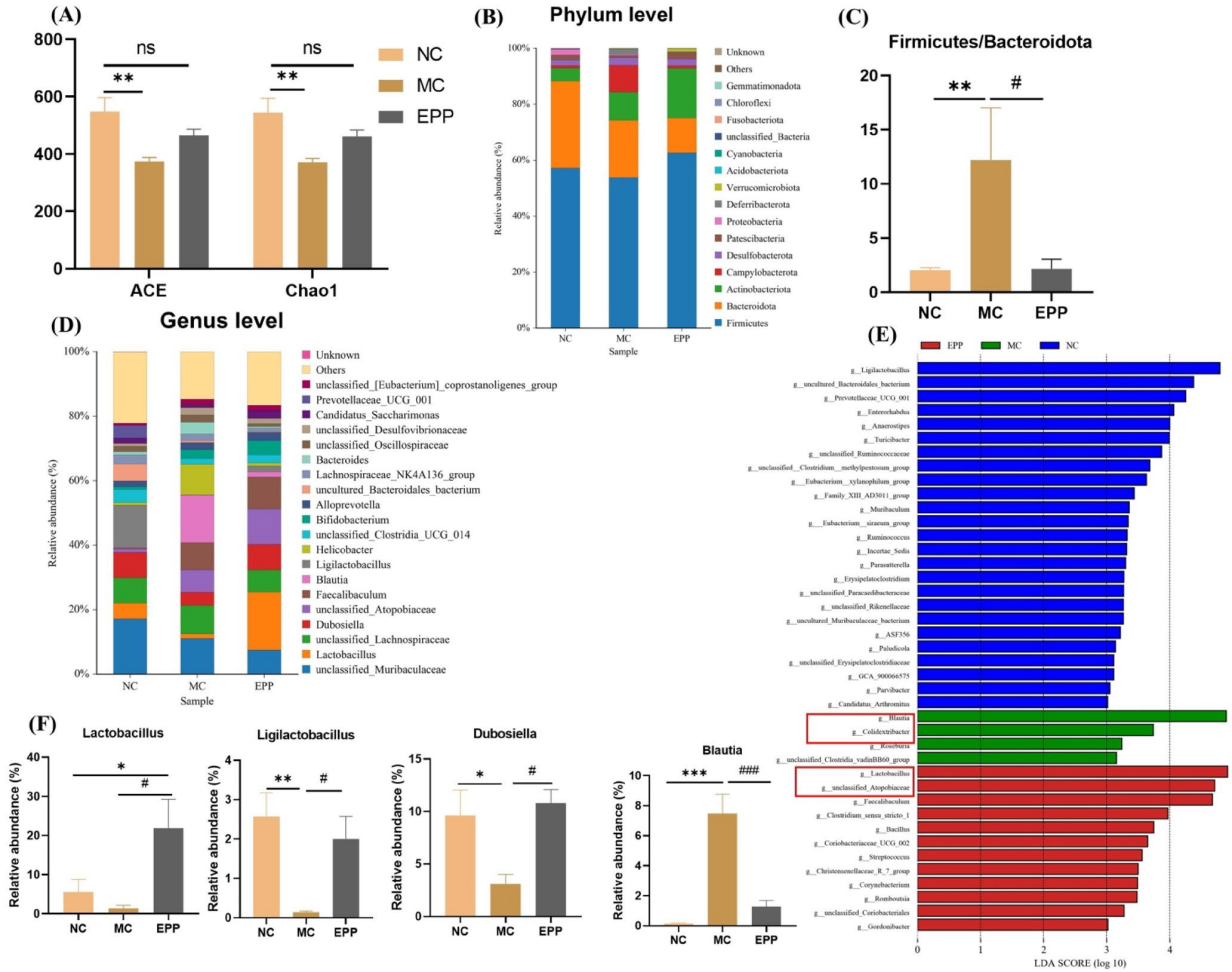
Effects of EPP on the composition of gut microbiota. (A) Alpha diversity indices (ACE and Chao1 indices). (B) Relative abundance of gut microbiota at the phylum level. (C) The ratio of *Firmicutes* to *Bacteroidetes* (F/B ratio). (D) Relative abundance of gut microbiota at the genus level. (E) Linear discriminant analysis (LDA) effect size (LEfSe) analysis (LDA score > 3.0, *p* < 0.05). (F) Relative abundance of four key differential genera. NC, normal control; MC, model control. *Statistical analysis:* Data are expressed as mean ± SEM (*n* = 6). Significance was determined by one‐way ANOVA with Tukey's post hoc test. For microbiome, metabolomics, and correlation analyses, *p*‐values were adjusted using the Benjamini‐Hochberg (FDR) method, with *q* < 0.05 considered significant. LEfSe used LDA score > 3.0 and *p* < 0.05. Compared with the NC group, *p < 0.05, **p < 0.01, ****p < 0.0001; compared with the MC group, ^#^ *p* < 0.05, ^###^ *p* < 0.001.

To investigate the effects of EPP on gut microbiota, we performed 16S rRNA gene sequencing to systematically analyze the impact of EPP on the diversity and composition of gut microbiota in diabetic mice. Alpha diversity analysis revealed a significant decrease in microbial species richness in the MC group compared to the NC group (Figure 3A, *p* < 0.01), while EPP intervention effectively restored microbial richness (Figure 3A, *p* = ns). Beta diversity analyses (PCoA and PLS‐DA) demonstrated distinct separation among the NC, MC, and EPP groups, with the EPP group clustering closer to the NC group (Figure S1A,B), suggesting that EPP ameliorates diabetes‐induced gut microbial dysbiosis.

At the phylum level, the predominant bacterial communities were Firmicutes, Bacteroidota, Actinobacteriota, and Campylobacterota (Figure 3B). Compared with the control group, the diabetic model group exhibited a significant increase in the Firmicutes/Bacteroidota ratio (Figure 3C, *p* < 0.01). However, EPP intervention restored this ratio to normal levels (Figure 3C, *p* < 0.05). Genus‐level analysis identified unclassified*_Muribaculaceae*, *Lactobacillus*, unclassified*_Lachnospiraceae*, *Ligilactobacillus*, and *Dubosiella* as the most abundant genera across all groups (Figure 3D). LEfSe analysis revealed group‐specific biomarkers (Figure 3E): the NC group was characterized by *g__Ligilactobacillus* and *g__*uncultured*_Bacteroidales_bacterium*; the MC group by *g__Blautia* and *g__Colidextribacter*; while the EPP group showed significant enrichment of *g__Lactobacillus* and *g__unclassified_Atopobiaceae*.

As shown in Figure 3F, compared to the NC group, the MC group exhibited significantly reduced relative abundances of *Ligilactobacillus* (*p* < 0.01) and *Dubosiella* (*p* < 0.05), along with a marked increase in *Blautia* abundance (*p* < 0.001). EPP treatment restored the abundances of these genera (*Ligilactobacillus*, *Dubosiella*, and *Blautia*) to levels comparable to the NC group (Figure 3F). Notably, the relative abundance of *Lactobacillus* in the EPP group was significantly higher than in both the NC and MC groups (*p* < 0.05), indicating that EPP specifically promotes the proliferation of probiotic bacteria.

### 3.5 WGCNA Analysis Reveals Gut Microbiota‐Mediated Regulation of Liver Metabolism by EPP

This study employed untargeted metabolomics to comprehensively investigate the effects of EPP on hepatic metabolic profiles in T2DM mice. Multivariate statistical analysis (PCoA and PCA) demonstrated distinct metabolic pattern separations among NC, MC, and EPP‐treated groups (Figure S2A,B), indicating that EPP intervention significantly altered hepatic metabolic characteristics in diabetic mice. LC–MS/MS analysis identified 1358 differentially expressed metabolites, with 160 significantly altered metabolites (40 upregulated and 120 downregulated) in the EPP group compared to the MC group (Figure S2C).

**FIGURE 4:**
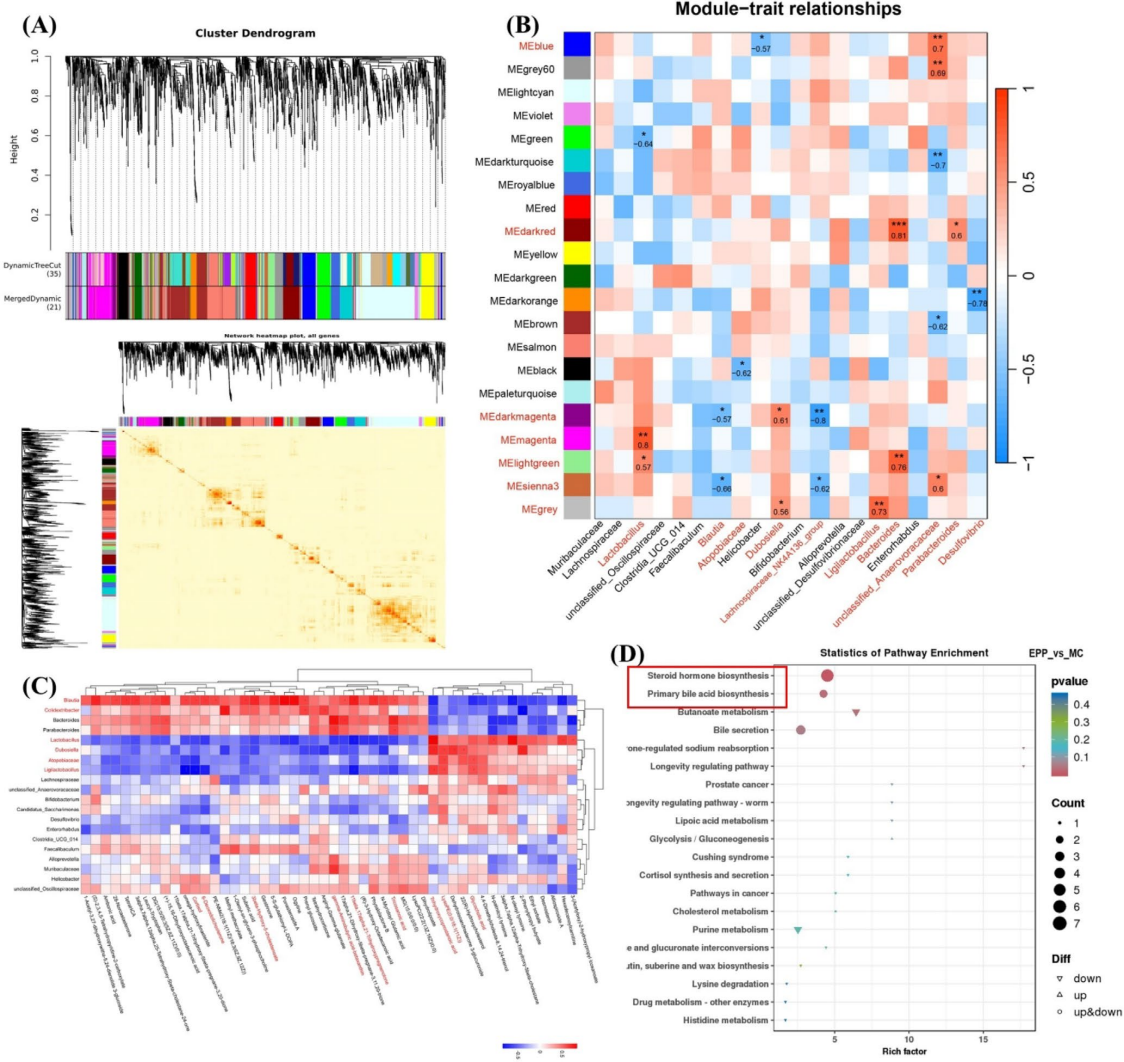
WGCNA and correlation analysis between hepatic metabolites and gut microbiota. (A) Clustering dendrogram of hepatic metabolites based on adjacency‐based dissimilarity, with assigned module colors and the corresponding topological overlap matrix heatmap. (B) Module‐trait relationship heatmap showing correlations between metabolite modules and bacterial genera. (C) Heatmap of Pearson correlation analysis between differential microbial genera and hepatic metabolites. Red represents a positive correlation, and blue represents a negative correlation. Key bacterial genera are highlighted in red. (D) KEGG enrichment bubble plot of the top 20 metabolic pathways enriched by differential metabolites. *Statistical analysis:* For (B) and (C), correlation *p*‐values were adjusted using the Benjamini‐Hochberg (FDR) method. For (D), KEGG enrichment significance was determined by a hypergeometric test with FDR correction (*q* < 0.05). Significant associations are marked by *p < 0.05, **p < 0.01, ***p < 0.001.

As depicted in Figure 4, we employed weighted gene co‐expression network analysis (WGCNA) to identify co‐expressed metabolic modules and investigate their associations with bacterial genera in liver samples. Network topology analysis determined a soft‐thresholding power of 9 as the optimal parameter, achieving a scale‐free topology fit index of 0.80 while maintaining adequate mean connectivity (Figure S3). Hierarchical clustering of hepatic metabolic profiles identified 21 distinct co‐expression modules, each represented by unique color codes (Figure 4A,B). The module‐trait relationship analysis revealed significant associations (*p* < 0.05) between 10 bacterial genera and 13 metabolic modules (Figure 4B). Notably, five modules (MEblue, MEdarkred, MEdarkmagenta, MElightgreen, and MEgrey) exhibited particularly strong correlations (|*r*| ≥ 0.7, *p* < 0.01) with specific genera, including *Lactobacillus*, *Colidextribacter*, *Ligilactobacillus*, *Bacteroides*, and *Atopobiaceae*. EPP supplementation significantly increased the relative abundance of both *Lactobacillus* and *Ligilactobacillus* (Figure 3F), consistent with their strong module associations (Figure 4B). Furthermore, the MEdarkmagenta module showed significant negative correlation with *Blautia* (*r* = −0.57, *p* < 0.05) and positive correlation with *reveals gut microbiota‐mediated regulation* (*r* = 0.61, *p* < 0.05). These findings collectively suggest that eight bacterial genera, particularly the differentially abundant *Lactobacillus*, *Ligilactobacillus*, *Blautia*, and *Dubosiella*, are strongly associated with EPP‐induced modifications in hepatic metabolism.

Pearson correlation analysis between the top 20 bacterial genera and 50 most significantly altered hepatic metabolites identified 40 differentially abundant metabolites that were significantly associated (*p* < 0.05) with these microbial taxa (Figure 4C). Intriguingly, enrichment analysis revealed that seven of these metabolites were involved in steroid hormone biosynthesis pathways (23.33%, Figure S2D). Specifically, trihydroxycoprostanoic acid and glycocholic acid, both significantly downregulated in the EPP‐treated group, were enriched in primary bile acid biosynthesis and cholesterol metabolism pathways. Additionally, cortisol, tetrahydrocortisone, 3β‐hydroxy‐5‐cholestenoate, and desmosterol, all classified as “steroids and steroid derivatives”, were enriched in steroid hormone biosynthesis pathways and showed significant correlations (*p* < 0.05) with multiple EPP‐modulated bacterial genera (Figure 4D). These results indicate that EPP intervention alters specific gut microbiota populations that correlate with shifts in hepatic steroid and bile acid metabolism, further supporting the involvement of a potential gut–liver axis mechanism in alleviating T2DM metabolic dysregulation.

### 3.6 Multi‐Omics Integration Indicates EPP May Exert Hypoglycemic Activity by Regulating Cholesterol‐Related Pathways

**FIGURE 5:**
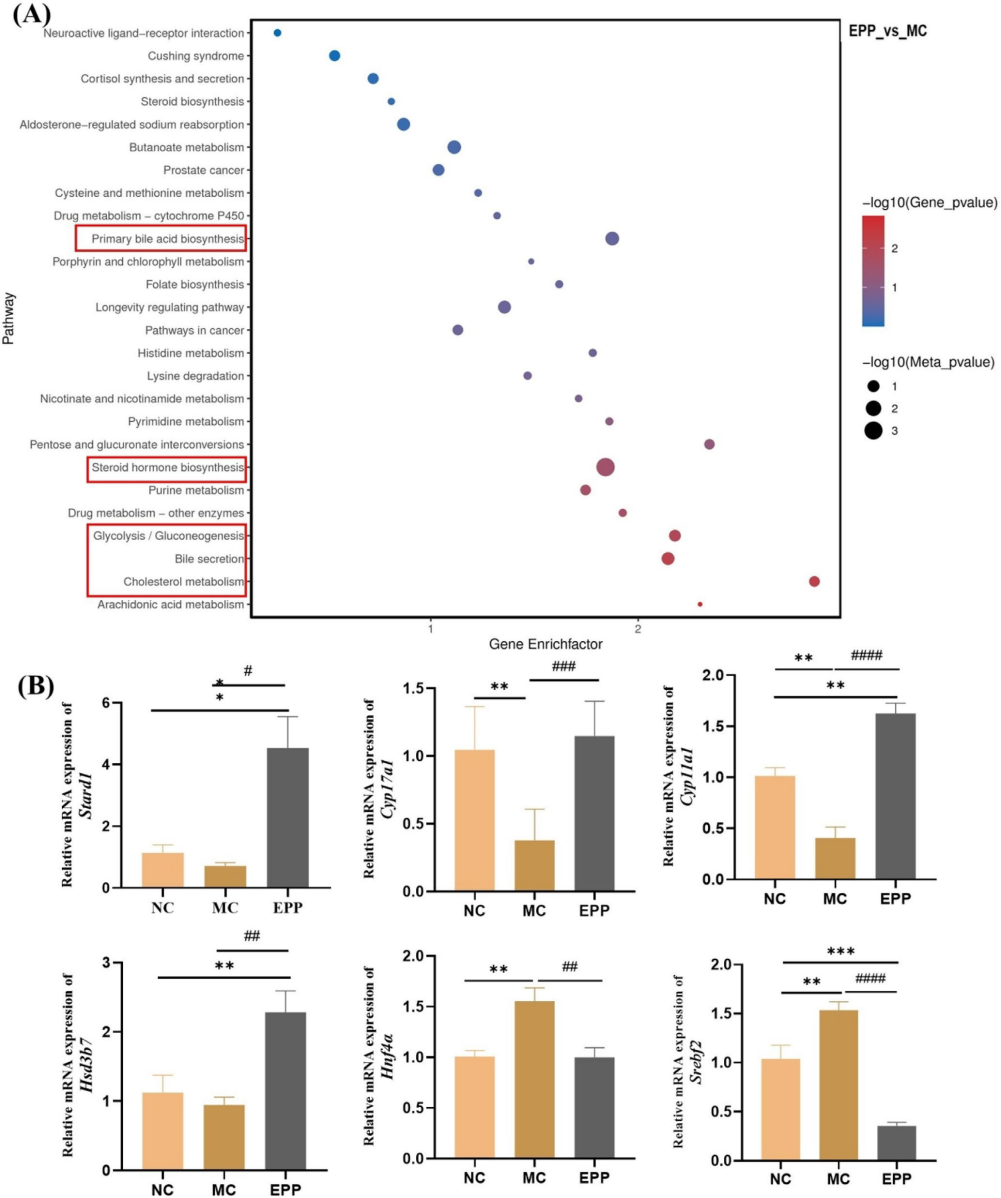
Integrative analysis of transcriptome and metabolome data with qPCR validation. (A) KEGG enrichment bubble plot of the top 30 pathways based on the joint analysis of differential genes and metabolites. (B) Relative mRNA expression levels of representative differential genes validated by qPCR. *Statistical analysis:* Data are presented as mean ± SEM (*n* = 6). For (A), KEGG joint enrichment significance was determined by a hypergeometric test with Benjamini‐Hochberg (FDR) correction. For (B), statistical significance of qPCR results was determined by one‐way ANOVA followed by Tukey's *post hoc* test. Relative expression was normalized to *β‐Actin*. Compared with the NC group, *p < 0.05, **p < 0.01, ***p < 0.001; compared with the MC group, ^#^ *p* < 0.05, ^##^ *p* < 0.01, ^###^ *p* < 0.001, ^####^ *p* < 0.0001.

To elucidate the hypoglycemic mechanism of EPP, we conducted an integrated analysis of liver transcriptomics and metabolomics data. RNA‐seq analysis revealed significant differential gene expression among the NC, MC, and EPP‐treated groups. Combined metabolomics analysis identified 29 significantly associated pathways shared between the two omics datasets (Figure S4A). By cross‐referencing the top 10 key nodes of gene pathways and metabolic pathways (Figure S4B) and screening the top 30 core pathways through KEGG enrichment analysis (Figure 5A), we found that EPP primarily regulates the following hypoglycemia‐related pathways: primary bile acid biosynthesis; steroid hormone biosynthesis; glycolysis/gluconeogenesis; bile secretion; and cholesterol metabolism. To validate the omics results, we examined the expression profiles of six hub genes (*Cyp17a1*, *Cyp11a1*, *Hsd3b7*, *Stard1*, *Srebf2*, and *Hnf4α*) via qPCR (Figure 5B), which showed highly consistent trends with the transcriptomic data (Figure 5B), confirming the reliability of the multi‐omics integrated analysis. These findings suggest that EPP may exert its hypoglycemic effects by modulating the “gene‐metabolite” interaction network, particularly pathways related to cholesterol metabolism.

### 3.7 Effects of EPP on SCFAs Levels and Their Spearman Correlation With Diabetes Pathological Markers and Gut Microbiota

**FIGURE 6:**
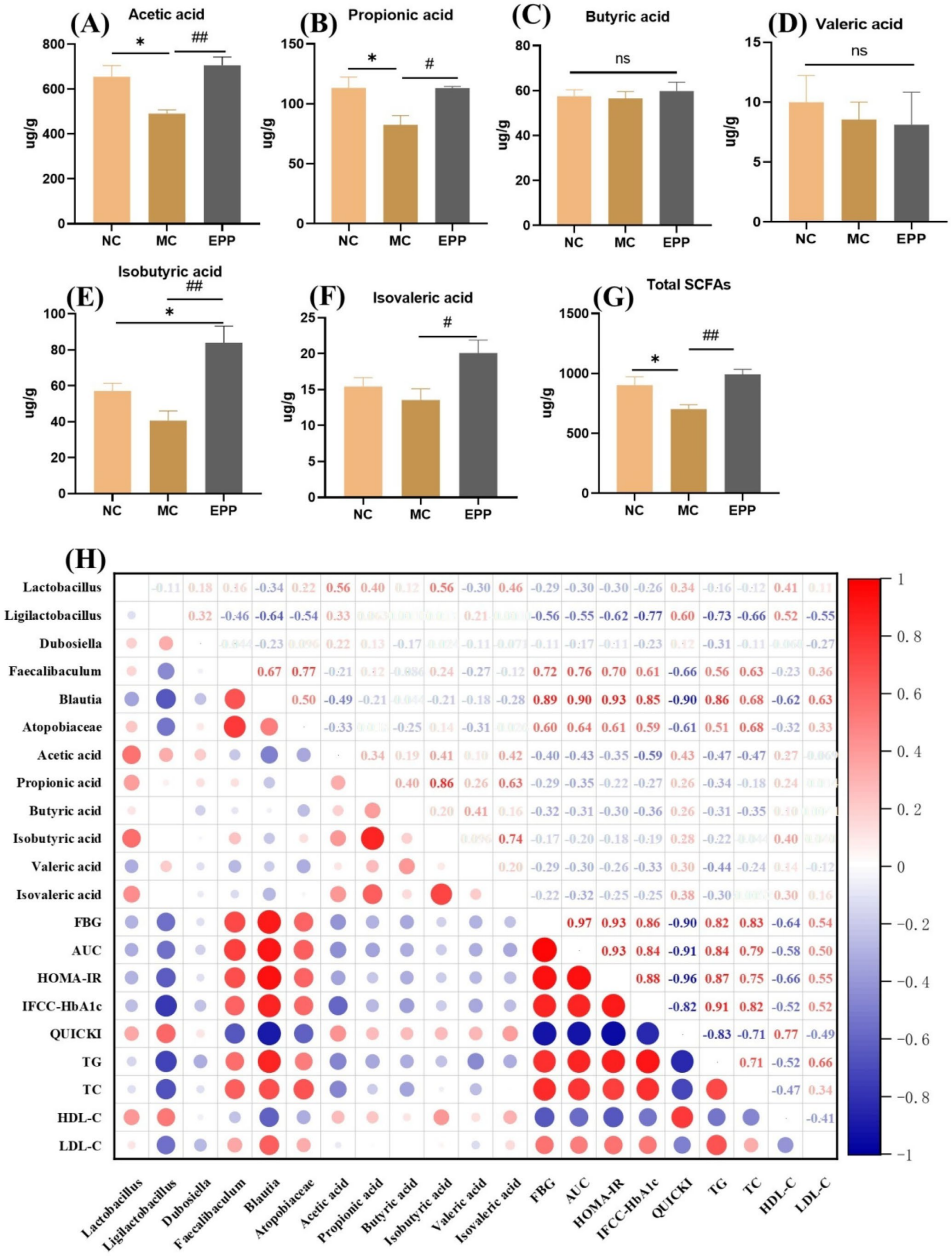
Effects of EPP on SCFAs levels and their spearman correlation with diabetes pathological markers and gut microbiota. (A) Acetic acid; (B) propionic acid; (C) butyric acid; (D) valeric acid; (E) isobutyric acid; (F) isovaleric acid; (G) total SCFAs. (H) Spearman correlation analysis. *Statistical analysis:* data are presented as mean ± SEM (*n* = 6). For (A–G), statistical significance of SCFA levels was determined by one‐way ANOVA followed by Tukey's post hoc test. For (H), spearman correlation *p*‐values were adjusted using the Benjamini‐Hochberg (FDR) method. Compared with the NC group, *p < 0.05; compared with the MC group, ^#^ *p* < 0.05, ^##^ *p* < 0.01.

As shown in Figure 6, compared with the NC group, the levels of acetic acid (Figure 6A, *p* < 0.05), propionic acid (Figure 6B, *p* < 0.05), and total SCFAs (Figure 6G, *p* < 0.05) in the feces of diabetic mice were significantly decreased. After EPP intervention, the levels of acetic acid (*p* < 0.01), propionic acid (*p* < 0.05), isobutyric acid (Figure 6E, *p* < 0.01), isovaleric acid (Figure 6F, *p* < 0.05), and total SCFAs (*p* < 0.01) in diabetic mice feces were significantly increased.

As shown in Figure 6H, spearman correlation analysis was employed to assess the correlations among 6 fecal SCFAs, 6 bacterial genera, and 9 T2DM‐related pathological indices. The results revealed that *Lactobacillus*, *Ligilactobacillus*, and *Dubosiella* showed negative correlations with FBG, AUC, HOMA‐IR, IFCC‐HbA1c, TG, TC, and LDL‐C, but positive correlations with QUICKI and HDL‐C. Conversely, *Faecalibaculum*, *Blautia*, and *Atopobiaceae* exhibited positive correlations with FBG, AUC, HOMA‐IR, IFCC‐HbA1c, TG, TC, and LDL‐C, and negative correlations with QUICKI and HDL‐C. All six SCFAs demonstrated negative correlations with FBG, AUC, HOMA‐IR, IFCC‐HbA1c, TG, TC, and LDL‐C, while showing positive correlations with QUICKI and HDL‐C. Furthermore, *Lactobacillus* was positively correlated with acetic acid, propionic acid, isobutyric acid, and isovaleric acid; *Ligilactobacillus* and *Dubosiella* also showed positive correlations with acetic acid. These findings suggest that EPP intervention may ameliorate diabetes‐related metabolic dysregulation through modulation of specific gut microbiota and their associated SCFA production.

## 4 Discussion

T2DM is a serious chronic metabolic disorder characterized by hyperglycemia and dyslipidemia. Prolonged hyperglycemia and hyperlipidemia can cause significant damage to various organs and tissues. Therefore, regulating blood glucose and lipid levels is crucial for the treatment and prevention of T2DM. Natural bioactive polysaccharides have been proposed as potential alternative therapeutic agents for T2DM. In this study, we established a T2DM mouse model induced by a high‐fat, high‐sugar diet combined with STZ administration to investigate the anti‐hyperglycemic effects of EPP. Evaluation of multiple physiological parameters demonstrated that EPP exerts beneficial effects on key aspects of T2DM. To explore the hypoglycemic mechanisms of EPP, we performed comprehensive analyses of the gut microbiome, metabolome, and transcriptome.

The fundamental characteristics of T2DM include elevated FBG levels, impaired glucose tolerance, insulin resistance, and dyslipidemia (Hassan et al. 2015). In our study, compared to the NC group, the MC group exhibited significantly higher FBG levels and OGTT values at all measured time points, whereas EPP intervention markedly reversed these increases. Furthermore, EPP effectively ameliorated dyslipidemia, consistent with previous findings (Lin et al. 2024), suggesting its potential benefit in diabetes prevention and management. HOMA and QUICKI, derived from fasting glucose and insulin levels, serve as established indices for assessing β‐cell function and insulin resistance (Cheng et al. 2020). Serum insulin analysis revealed that EPP significantly reduced the HOMA‐IR index while increasing the QUICKI index in diabetic mice, aligning with prior observations for tea‐derived polysaccharides (H. Li et al. 2020). These findings strongly indicate that oral EPP administration may represent an effective strategy for lowering blood glucose levels.

Excessive and reversible fat accumulation in the liver has been identified as a major contributor to T2DM (Taylor et al. 2019). In this study, histopathological analysis of liver tissue staining revealed that EPP treatment restored normal hepatic microstructure morphology, suggesting its potential protective effect against diabetes‐associated liver injury. Dyslipidemia, a common comorbidity in diabetic patients, is characterized by elevated TC, TG, and LDL‐C levels alongside reduced HDL‐C (Ajeigbe et al. 2022). Thus, lipid biomarkers—including TC, TG, LDL‐C, and HDL‐C—serve as critical diagnostic indicators for dyslipidemia. Our findings demonstrate that EPP significantly decreased serum TC, TG, and LDL‐C while increasing HDL‐C levels. Collectively, these results indicate that EPP improves glucose regulation by ameliorating dyslipidemia and mitigating hepatic damage.

In recent years, accumulating evidence has demonstrated a close association between gut microbiota dysbiosis and the pathogenesis of various metabolic disorders, including metabolic syndrome, obesity, T2DM, and cardiovascular diseases (J. Zhang 2024). Particularly, germ‐free animal models and fecal microbiota transplantation studies have provided compelling evidence for the causal role of gut microbiota in these metabolic diseases, establishing it as a promising therapeutic target (Wu et al. 2023). Notably, dietary polysaccharides have emerged as potential interventions for T2DM due to their ability to effectively modulate gut microbiota dysbiosis in diabetic mice (Xue et al. 2024). Our study employing PCoA and PLS‐DA revealed significant gut microbiota dysbiosis in diabetic mice. At the phylum level, Firmicutes and Bacteroidetes were identified as the predominant microbial populations. Importantly, an elevated Firmicutes‐to‐Bacteroidetes (F/B) ratio has been well‐documented as a key factor contributing to metabolic disorders and insulin resistance (Hills Jr. et al. 2019). Remarkably, EPP intervention significantly reduced the relative abundance of Firmicutes and the F/B ratio, suggesting its potential to ameliorate metabolic disorders through modulation of this critical microbial signature. Further analysis demonstrated that EPP treatment specifically enriched beneficial bacterial taxa (*Lactobacillus*, *Ligilactobacillus*, and *Dubosiella*) that were diminished by high‐sugar high‐fat/streptozotocin treatment, while simultaneously reducing the relative abundance of *Blautia*. *Lactobacillus* and *Ligilactobacillus*, as important probiotics in the human gastrointestinal tract, not only inhibit opportunistic pathogens associated with metabolic diseases but also exert natural anti‐diabetic effects by remodeling gut microbial composition (Mao et al. 2024). *Dubosiella* has been closely associated with the pathogenesis of various metabolic disorders, particularly diabetes mellitus (Jianpeng Li et al. 2022). Notably, certain *Dubosiella* strains have been clinically utilized as probiotic supplements due to their beneficial metabolic effects (T.‐h. Liu et al. 2021). As demonstrated by Qiu et al., specific *Dubosiella* species can effectively modulate SCFAs production, thereby attenuating obesity progression through improved metabolic regulation (Qiu et al. 2021). In contrast, *Blautia* has been consistently reported to show positive correlation with T2DM in multiple studies (Gurung et al. 2020). Collectively, these results suggest that EPP may ameliorate metabolic disorders by modulating gut microbiota composition through promoting beneficial bacterial proliferation while suppressing harmful bacterial growth.

The therapeutic potential of *Lactobacillus* in T2DM management has been extensively documented. For instance, Zhao et al. demonstrated that polysaccharides from *Onchidium struma* ameliorated diabetic symptoms and hepatic lipid accumulation by modulating *Lactobacillus* abundance and increasing fecal SCFA levels (Zhao et al. 2023). Similarly, Zhang et al. reported that polysaccharides from Fu brick tea restored gut microbiota balance in T2DM rats by enriching beneficial taxa (*Ruminococcus*, *Lactobacillus*, and *Lachnospiraceae_NK4A136_group*) while suppressing detrimental genera (*Prevotella* and *Coprococcus*) (X. Zhang et al. 2023). Complementary evidence from Chen et al. further confirmed that mulberry polysaccharides improved lipid profiles (TC, TG, LDL‐C, and HDL‐C) by regulating *Lactobacillus*, *Allobaculum*, *Bacteroides*, and *Akkermansia* (C. Chen et al. 2018). Collectively, these studies indicate that although different polysaccharides target distinct microbial taxa, their shared ability to modulate gut microbiota and alleviate T2DM symptoms underscores a consistent therapeutic mechanism. This mechanistic consistency suggests that EPP's antidiabetic effects may similarly involve the promotion of beneficial bacteria (particularly *Lactobacillus*) and improvement of glucose/lipid metabolism. *Lactobacillus* species contribute to these effects through their enzymatic capacity to hydrolyze dietary polysaccharides into SCFAs via carbohydrate‐active enzymes (CAZymes) (Gao et al. 2018). SCFAs, in turn, mediate metabolic benefits by serving as both energy substrates for colonocytes and signaling molecules that activate G protein‐coupled receptors, thereby regulating glucose homeostasis and insulin sensitivity (Portincasa et al. 2022). Specifically, acetate enhances insulin secretion and sensitivity, propionate and butyrate stimulate intestinal gluconeogenesis, and isovalerate may influence glycemic control through microbial modulation (Song et al. 2025). Notably, these gut‐derived SCFAs can be transported via the portal vein to the liver, where they potentially act as signaling molecules to regulate the expression of key hepatic metabolic genes (Mukhopadhya and Louis 2025). This portal transport mechanism establishes a direct functional link between EPP‐induced microbial fermentation and hepatic transcriptional reprogramming. These findings align with prior reports demonstrating that polysaccharides from *Phellinus linteus* and *Ascosphaera* improve metabolic health via SCFA‐dependent mechanisms (L. Chen et al. 2023; T. Liu et al. 2024). Given this evidence, we propose that EPP exerts its antidiabetic effects through microbiota‐mediated SCFA production.

To elucidate the molecular mechanisms underlying EPP‐mediated amelioration of diabetic phenotypes, this study conducted an integrated analysis of metabolomics and transcriptomics. Metabolomic profiling revealed significant enrichment of key metabolic pathways in the EPP treatment group, including steroid hormone biosynthesis, primary bile acid biosynthesis, butanoate metabolism, and bile secretion. Given the central role of the liver in regulating systemic glucose and lipid homeostasis, it exerts precise control over these balances through critical metabolic pathways such as gluconeogenesis, glycolysis, lipogenesis, cholesterol metabolism, and fatty acid oxidation (T. Li and Chiang 2024). Within the hepatic cholesterol metabolism network, a hierarchical and synergistic interplay of key genes orchestrates this process: Steroidogenic acute regulatory protein (StARD1) facilitates the transport of cholesterol from the outer to the inner mitochondrial membrane, supplying substrate for subsequent metabolism (Larsen et al. 2020). Subsequently, cholesterol side‐chain cleavage enzyme (CYP11A1) catalyzes the cleavage of the cholesterol side chain within the mitochondria, generating pregnenolone—the initial step in steroid hormone synthesis. Cytochrome P450 17A1 (CYP17A1) then hydroxylates pregnenolone to yield precursors of glucocorticoids and sex hormones (Miller and Bose 2011; Schiffer et al. 2019). Concurrently, the rate‐limiting enzyme for bile acid synthesis, cholesterol 7α‐hydroxylase (CYP7A1), converts cholesterol to 7α‐hydroxycholesterol. In the alternative pathway, Hydroxysteroid (17‐beta) dehydrogenase 7 (HSD3B7) further catalyzes the synthesis of bile acid intermediates, effectively driving cholesterol catabolism (Chiang and Ferrell 2022). Results from qRT‐PCR analysis demonstrated that EPP significantly upregulated the hepatic mRNA expression levels of critical cholesterol metabolism genes, including *Cyp17a1*, *Cyp11a1*, *Hsd3b7*, and *Stard1*. Although protein‐level validation was not conducted, the observed transcriptional activation of the *Cyp7a1*/*Hsd3b7*‐mediated bile acid synthesis pathway is highly consistent with the significant reduction in systemic cholesterol and LDL‐C levels. In metabolic disease models, mRNA abundance of these rate‐limiting enzymes often serves as a reliable surrogate for enzymatic flux, providing strong evidence for the EPP‐mediated cholesterol‐to‐bile acid metabolic shunt. Furthermore, EPP intervention significantly downregulated the mRNA expression levels of *Srebf2* and *Hnf4α*, key transcriptional regulators of cholesterol synthesis and gluconeogenesis. SREBP‐2, a sterol‐sensitive transcription factor, maintains cholesterol homeostasis by positively regulating the expression of genes involved in cholesterol biosynthesis and uptake (X. Li et al. 2021). Elevated expression of SREBP‐2 has been confirmed in high‐fat diet‐induced obese rat models (Khaleel et al. 2018), and its overexpression exacerbates lipid deposition and upregulates associated metabolic genes (Malhotra et al. 2017). HNF4α, a highly conserved transcription factor belonging to the nuclear receptor superfamily, plays a pivotal role in regulating hepatocyte differentiation, growth, and function (Xin Xu et al. 2020). Studies indicate that HNF4α directly activates the transcription of key gluconeogenic enzymes, glucose‐6‐phosphatase (G6Pase) and phosphoenolpyruvate carboxykinase (PEPCK), by binding to its cognate cis‐regulatory elements within their promoter regions (M. Zhang et al. 2012). Its critical role in diabetes pathogenesis has been definitively established by the HNF4α G115S mutation, which disrupts β‐cell function through loss of transcriptional activity (Tavares‐Sanchez et al. 2015). Consequently, targeted inhibition of HNF4α has emerged as a novel therapeutic strategy for T2DM. Several HNF4α‐targeting compounds, such as the flavonoids luteolin and 4‐nitro‐6‐hydroxyflavone, have been identified that improve glucose homeostasis in model organisms (Inoue et al. 2017; Juan Li et al. 2015).

Furthermore, the gut microbiota plays a crucial role in maintaining host cholesterol homeostasis. Numerous bacterial genera possess the ability to metabolize cholesterol, and this study found that EPP intervention significantly increased the abundance of *Lactobacillus* and *Dubosiella* in the mouse gut. Notably, these genera predominantly consist of bile salt hydrolase (BSH)‐positive strains (B. V. Jones et al. 2008), suggesting that EPP may influence the host's bile acid metabolic profile by modulating the gut microbiota. Extensive studies have demonstrated that BSH‐active probiotic bacteria can regulate host cholesterol metabolism through multiple mechanisms. For instance, Jones et al. confirmed that probiotics with elevated BSH activity significantly reduced serum LDL‐C, indicating a direct association between BSH activity and host cholesterol regulation (M. L. Jones, Martoni, and Prakash 2012). Similarly, Wang et al. reported in a human trial that supplementation with the highly BSH‐active strain *Lactobacillus reuteri* NCIMB 30242 decreased serum LDL‐C and reduced plasma phytosterol levels, suggesting inhibition of intestinal cholesterol absorption (Mitchell L. Jones, Martoni, Parent, and Prakash 2012). Moreover, BSH‐mediated bile acid deconjugation promotes the fecal excretion of free bile acids, thereby enhancing hepatic cholesterol consumption (for de novo bile acid synthesis) and further lowering systemic cholesterol levels (Joyce et al. 2019). Joyce et al. demonstrated that *Lactobacillus salivarius* expressing high BSH activity significantly reduced LDL‐C, total cholesterol, and triglyceride levels in mice (Joyce et al. 2014).

Building upon the established structural characteristics and general hypoglycemic effects of EPP (Yuan et al. 2019; Zhu et al. 2023), the systemic molecular network underling these effects remains to be fully elucidated. Our data suggest that EPP intervention is associated with the recruitment of BSH‐active taxa (e.g., *Dubosiella* and *Lactobacillus*), which appears to synchronize with the transcriptional activation of key hepatic cholesterol catabolism genes (*Cyp11a1*, *Stard1*, and *Hsd3b7*). This evidence chain offers a plausible link between gut microbial functionality and hepatic transcriptional responses, providing a more comprehensive perspective on EPP's systemic effects. Despite these mechanistic insights, several limitations of the current study should be acknowledged. First, while the activation of the cholesterol‐to‐bile acid metabolic pathway was validated at the transcriptional level using qRT‐PCR, further Western blotting is warranted to confirm these findings at the protein level, as post‐transcriptional modulations may influence actual enzymatic expression and activity. Second, although our multi‐omics data point to a potential link involving bile acid metabolism, future validation via fecal microbiota transplantation and targeted metabolomics to quantitatively measure specific bile acid profiles in the serum, liver, and feces would provide more direct, causal evidence. Finally, as the EPP used here is a macromolecular polysaccharide, further purification and structural characterization of homogeneous fractions are needed to precisely identify the primary bioactive structures and establish clearer structure–activity relationships. Nevertheless, this integrated analysis provides a solid foundation for understanding the systemic benefits associated with EPP and offers valuable insights for developing polysaccharide‐based dietary interventions for T2DM management.

## 5 Conclusion

This study suggests that EPP potentially alleviates T2DM, which is closely associated with changes in the gut microbiota–liver axis and cholesterol metabolism. The major novelty lies in providing a prospective mechanistic framework linking EPP‐mediated gut microbiota alterations to hepatic cholesterol homeostasis via bile acid synthesis. This proposed mechanism is supported by integrated microbiome, metabolome, and transcriptome analyses, revealing tight correlations between gut microbial shifts and hepatic transcriptional reprogramming. Specifically, EPP was associated with the enrichment of beneficial bacteria (*Lactobacillus*, *Dubosiella*), enhanced SCFA production, and the altered expression of cholesterol/bile acid metabolism‐related genes (*Cyp11a1*, *Hsd3b7*, *Stard1*, *Srebf2*, *Hnf4α*). These findings support the potential of EPP as a prospective dietary supplement for T2DM management, though further protein‐level validation and targeted bile acid profiling remain warranted to fully elucidate the underlying causal pathways.

## Author Contributions

**Liuying Zhu:** writing – original draft, validation, methodology, investigation, formal analysis, data curation, conceptualization. **Xueqian Hu:** software, methodology, writing – review and editing. **Xusheng Wen:** methodology, investigation, data curation, conceptualization. **Yanqun Xu:** writing – review and editing, methodology. **Wenrong Zhu:** supervision, resources, project administration, funding acquisition. **Xin Peng:** writing – review and editing, funding acquisition, methodology, supervision, resources, project administration.

## Funding

This work was supported by Ningbo Top Medical and Health Research Program (2022030309), Major Research and Development Plan Project (2023Z118), “Pioneer” and “Leading Goose” R&D Program of Zhejiang (2023C03038), National Administration of TCM and Zhejiang Province (GZY‐ZI‐KJ‐23037). Ningbo Major Research and Development Plan Project 2022Z135.

## Ethics Statement

The present study was ethically approved by the Animal Ethics Committee of Guoke Ningbo Life Science and Health Industry Research Institute under the code of GK‐2025‐XM‐0003.

## Conflicts of Interest

The authors declare no conflicts of interest.

## Supporting information


**Table S1:** Primer sequences.
**Figure S1:** Effects of EPP on the composition of gut microbiota with beta diversity analyses. (A) Principal coordinates analysis (PCoA); (B) partial least squares discrimination analysis (PCA).
**Figure S2:** Comparative analysis of liver metabolomics analysis in MC group and EPP‐treated groups. (A) Principal coordinates analysis (PCoA); (B) principal component analysis (PCA). (C) Differential metabolite statistical chart; (D) the KEGG functional annotation of differential metabolites.
**Figure S3:** Network topology analysis for different soft threshold powers.
**Figure S4:** Integrated analysis of liver transcriptomics and metabolomics. (A) Venn diagram of differential genes and differential metabolite pathways; (B) the top 10 pathways with the most differentially expressed genes/metabolites.

## Data Availability

The data that support the findings of this study are available from the corresponding author upon reasonable request. The multi‐omics sequencing data are available from the Sequence Read Archive (SRA) under accession number PRJNA1440651.
