# Supplementary material for: Enteromorpha prolifera Polysaccharide Alleviates Type 2 Diabetes via the Gut Microbiota–Liver Axis to Modulate Cholesterol Metabolism
Source: Food Sci Nutr. 2026 Jun 9;14(6):e71998. doi: 10.1002/fsn3.71998 (PMC13249803; doi:10.1002/fsn3.71998)
Supplement: Supplementary file 1 — Table S1: Primer sequences. Figure S1: Effects of EPP on the composition of gut microbiota with beta diversity analyses. (A) Principal coordinates analysis (PCoA); (B) partial least squares discrimination analysis (PCA). Figure S2: Comparative analysis of liver metabolomics analysis in MC group and EPP‐treated groups. (A) Principal coordinates analysis (PCoA); (B) principal component analysis (PCA). (C) Differential metabolite statistical chart; (D) the KEGG functional annotation of differential metabolites. Figure S3: Network topology analysis for different soft threshold powers. Figure S4: Integrated analysis of liver transcriptomics and metabolomics. (A) Venn diagram of differential genes and differential metabolite pathways; (B) the top 10 pathways with the most differentially expressed genes/metabolites. [file FSN3-14-e71998-s001.docx]

**Table S1 Primer sequences**

| Genes | Forward primer | Reverse primer |
| --- | --- | --- |
| *β-actin* | ACGGCCAGGTCATCACTATTG | TGGATGCCACAGGATTCCA |
| *Stard1* | 5'-ACGAGGGCTAGGGCCAAAT-3' | 5'-CAGACCCCTTATGCCTCCC -3' |
| *Cyp11a1* | 5′-CGAATCGTCCTAAACCAAGAG-3′ | 5′-CACTGATGACCCCTGAGAAAT-3′ |
| *Hsd3bF* | 5′-CAAGTGTGCCAGCCTTCATCT-3′ | 5′-TTCATGATTCTGTTCCTCGTGG-3′ |
| *Srebf2* | 5'-CAAGGCCATCGACTACATCCG-3' | 5′-CACCACTTCGGGTTTCATGC-3′ |
| *Cyp17a1* | 5'-CCAGGACCCAAGTGTGTTCT-3' | 5'-CCTGATACGAAGCACTTCTCG-3' |
| *Hnf4α* | 5'-ATGCTTCTCGGAGGGTCTGC-3' | 5'-GGAGTCTCGGGAGTGGACATCT-3' |


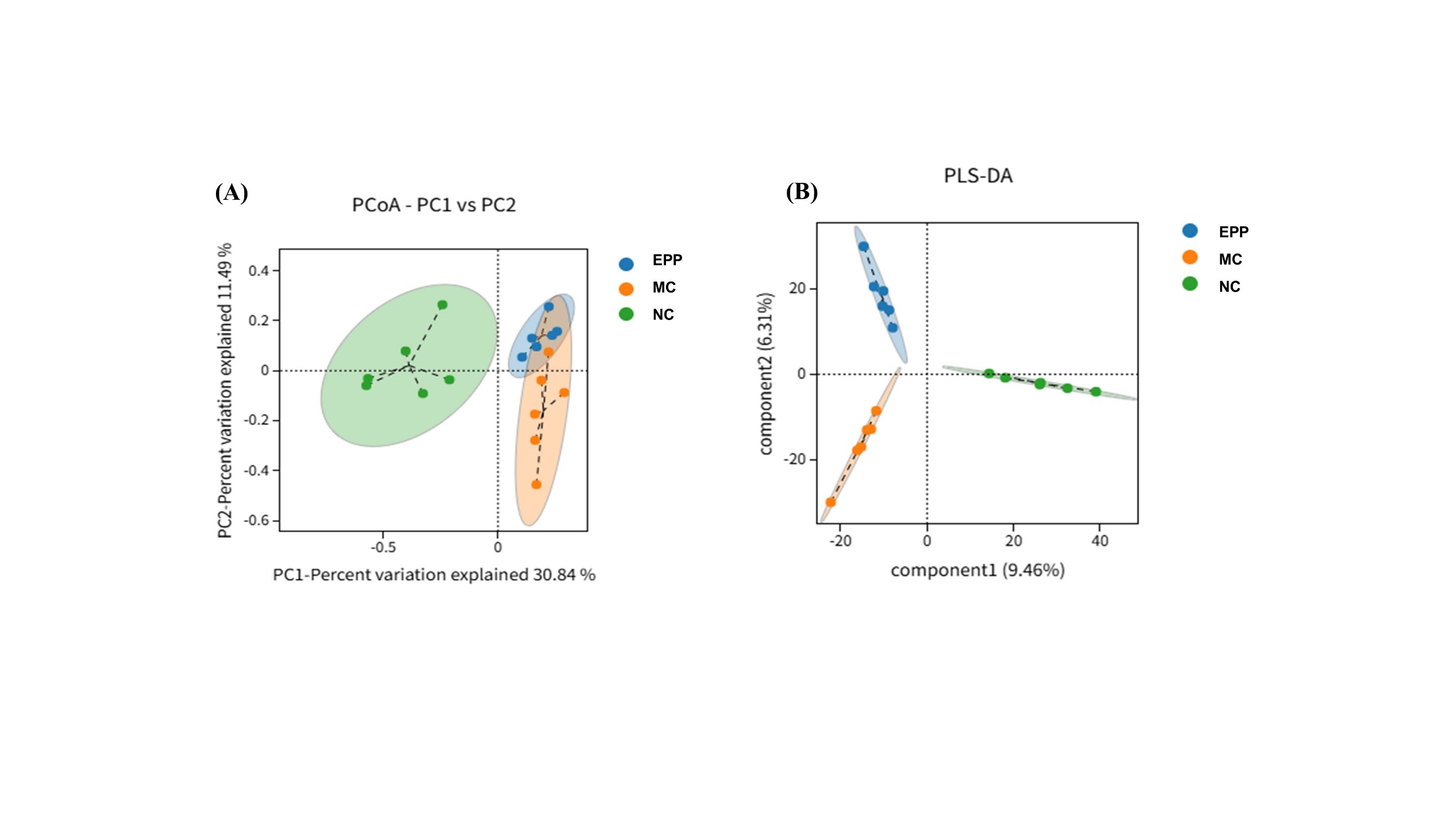


**Figure S1** **Effects of EPP on the composition of gut microbiota with beta diversity analyses.** (A) Principal coordinates analysis (PCoA); (B) Partial least squares discrimination analysis (PCA).


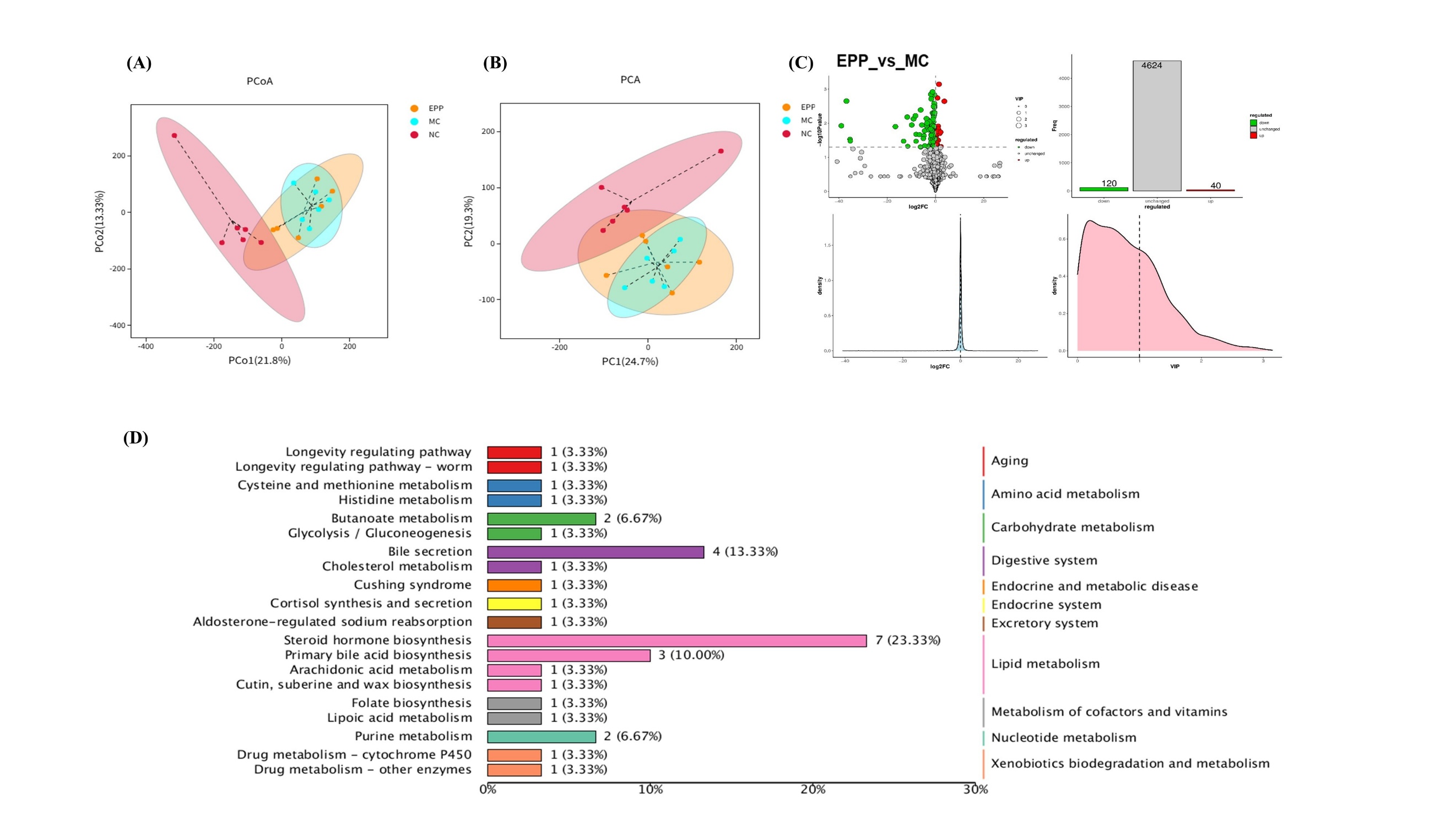
**Figure S2. Comparative analysis of liver metabolomics analysis in MC group and EPP-treated groups.** (A) Principal coordinates analysis (PCoA); (B) Principal component analysis (PCA). (C) Differential metabolite statistical chart; (D) The KEGG functional annotation of differential metabolites

**
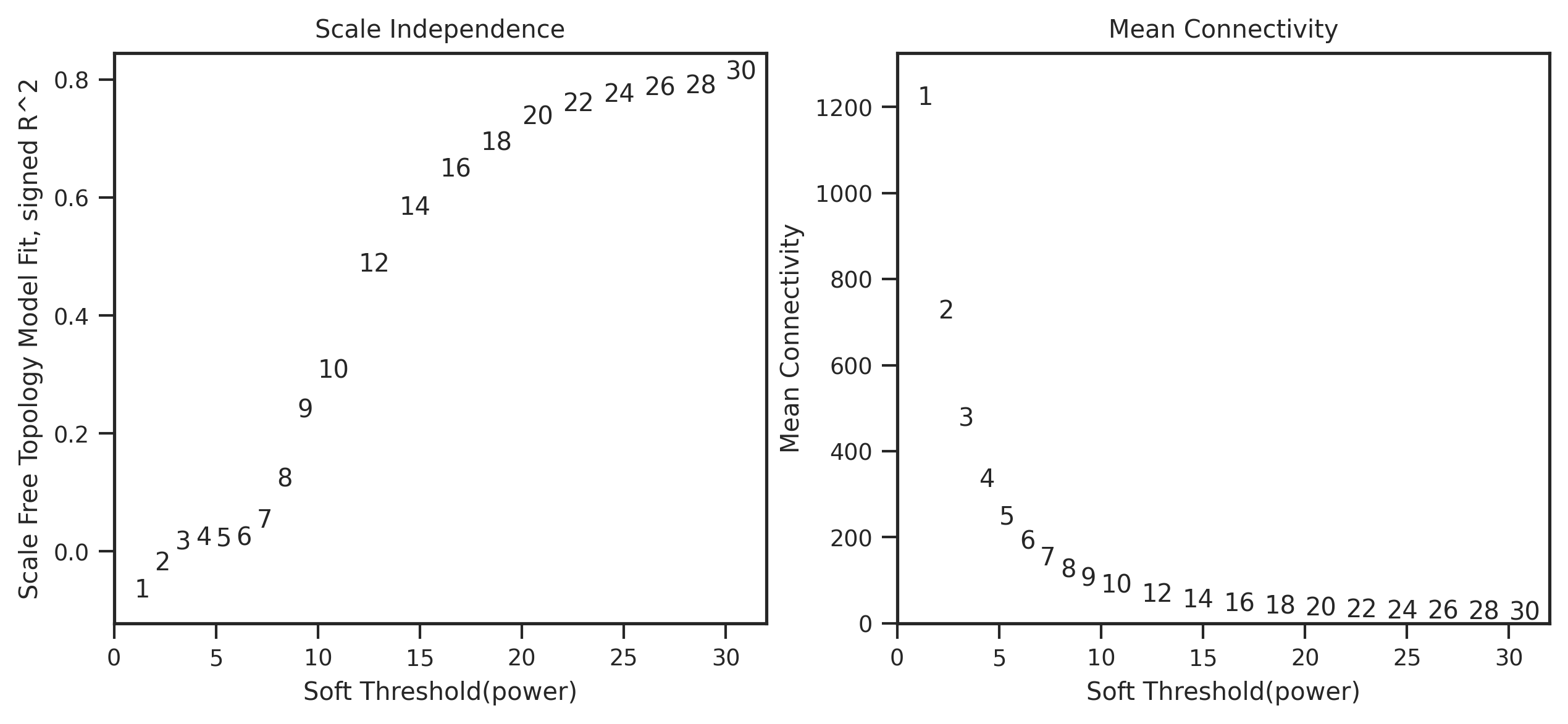
Figure S3. Network topology analysis for different soft threshold powers**.


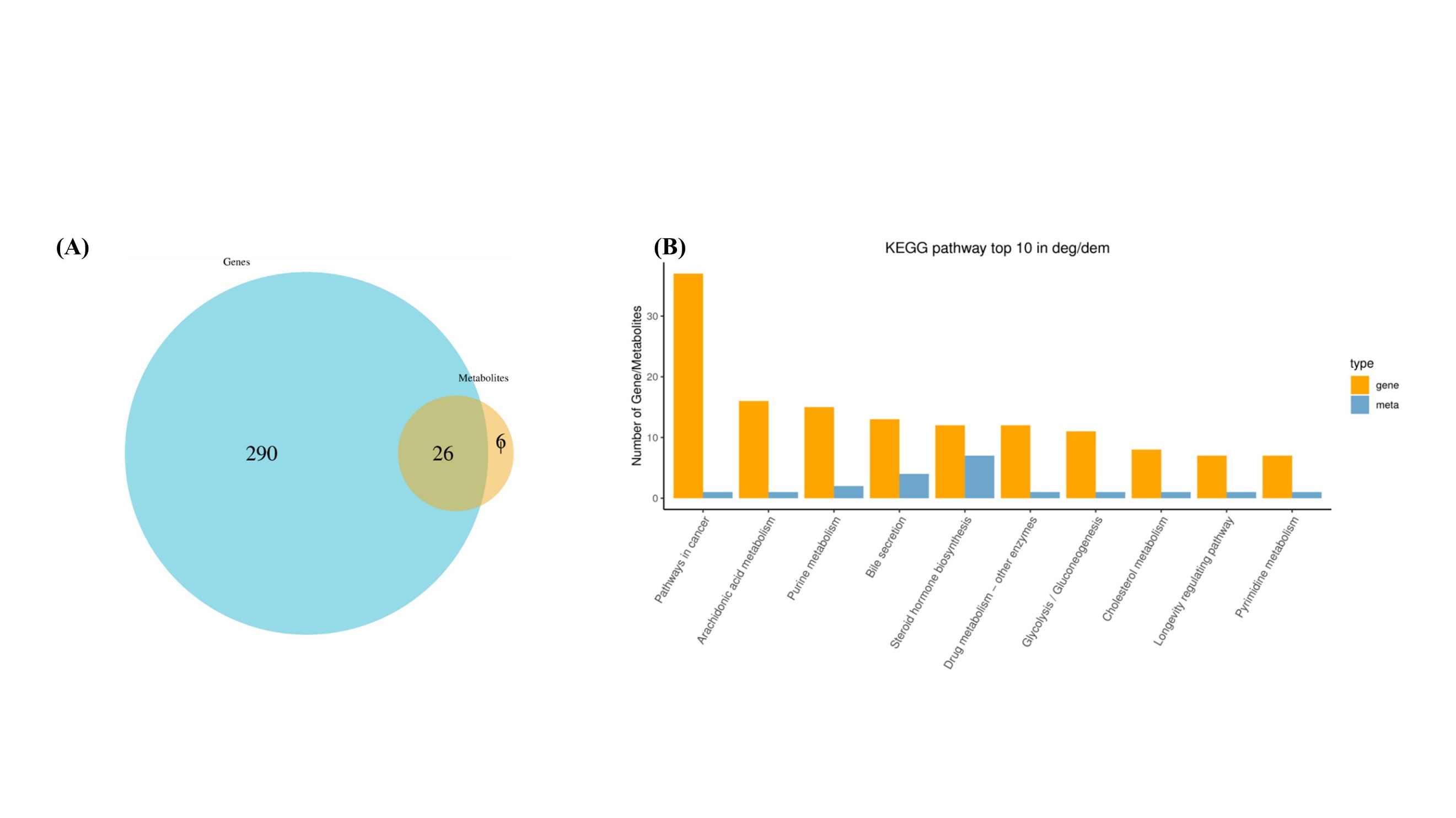


**Figure S4. Integrated analysis of liver transcriptomics and metabolomics.** (A) Venn diagram of differential genes and differential metabolite pathways; (B) The top 10 pathways with the most differentially expressed genes/metabolites.
